# Predicting mortality in acutely hospitalised older patients: the impact of model dimensionality

**DOI:** 10.1186/s12916-022-02698-2

**Published:** 2023-01-08

**Authors:** Alex Tsui, Petru-Daniel Tudosiu, Mikael Brudfors, Ashwani Jha, Jorge Cardoso, Sebastien Ourselin, John Ashburner, Geraint Rees, Daniel Davis, Parashkev Nachev

**Affiliations:** 1 MRC Unit for Lifelong Health and Ageing at UCL, London, UK; 2 School of Imaging and Biomedical Engineering, King’s College London, London, UK; 3 Wellcome Centre for Human Neuroimaging, UCL, London, UK; 4 UCL Queen Square Institute of Neurology, UCL, London, UK

**Keywords:** Mortality prediction, Geriatrics, Frailty, Multi-modal modelling, Brain imaging

## Abstract

**Background:**

The prediction of long-term mortality following acute illness can be unreliable for older patients, inhibiting the delivery of targeted clinical interventions. The difficulty plausibly arises from the complex, multifactorial nature of the underlying biology in this population, which flexible, multimodal models based on machine learning may overcome. Here, we test this hypothesis by quantifying the comparative predictive fidelity of such models in a large consecutive sample of older patients acutely admitted to hospital and characterise their biological support.

**Methods:**

A set of 804 admission episodes involving 616 unique patients with a mean age of 84.5 years consecutively admitted to the Acute Geriatric service at University College Hospital were identified, in whom clinical diagnoses, blood tests, cognitive status, computed tomography of the head, and mortality within 600 days after admission were available. We trained and evaluated out-of-sample an array of extreme gradient boosted trees-based predictive models of incrementally greater numbers of investigational modalities and modelled features. Both linear and non-linear associations with investigational features were quantified.

**Results:**

Predictive models of mortality showed progressively increasing fidelity with greater numbers of modelled modalities and dimensions. The area under the receiver operating characteristic curve rose from 0.67 (sd = 0.078) for age and sex to 0.874 (sd = 0.046) for the most comprehensive model. Extracranial bone and soft tissue features contributed more than intracranial features towards long-term mortality prediction. The anterior cingulate and angular gyri, and serum albumin, were the greatest intracranial and biochemical model contributors respectively.

**Conclusions:**

High-dimensional, multimodal predictive models of mortality based on routine clinical data offer higher predictive fidelity than simpler models, facilitating individual level prognostication and interventional targeting. The joint contributions of both extracranial and intracranial features highlight the potential importance of optimising somatic as well as neural functions in healthy ageing. Our findings suggest a promising path towards a high-fidelity, multimodal index of frailty.

**Supplementary Information:**

The online version contains supplementary material available at 10.1186/s12916-022-02698-2.

## Background

As a clinical outcome of the greatest concern, mortality demands predictive instruments of the highest fidelity: to guide expectations, target interventions, and illuminate modifiable mechanisms of disease [1]. Ordinarily narrowed to specific causes, it is also a general, constitutional risk in older patients, distributed across a wide causal field of biological and pathological factors, both incident and enduring. The determinants of such vulnerability may inhere less in any specific condition than in the complex interaction of multiple accruing co-morbidities and age-related physiological changes that single disease-centred models cannot satisfactorily capture. Predicting mortality here arguably requires a patient-centred, fully inclusive, yet population-scalable approach, capable of absorbing the wide heterogeneity of factors plausibly determining individual risk in older patients: the largest contingent of healthcare users, with the most variable intragroup functional and cognitive performance [2]. Though only 18% of the UK population, people aged ≥ 65 account for 42% of acute hospital admissions [3], a gap projected to widen as those over 60 are expected to double in number by 2050 worldwide [4].

Several short and medium-term mortality prediction instruments exist, such as APACHE-III [5], HELP [6], BISEP [7], SAFES [8], and HOMR [9]. Their applicability to acutely admitted unselected older patients is, however, limited by unproven generalisability beyond specific clinical settings such as critical care [5], poor calibration with age [10], variation in performance across population groups [11], dependence on background information not readily available in the acute setting [9], and use of features, such as clinical history and service utilisation, that are difficult to render objectively quantifiable and reproducible across healthcare systems. Crucially, current instruments rarely address two cardinal potential characteristics of the problem: the distribution of factors material to mortality across *multiple* clinical domains and investigational modalities, and the likely presence of heterogeneous causal interactions plausibly accessible only to *complex*, high-dimensional models. No survival instrument in current use attempts to draw power from the synthesis of multiple, individually weakly predictive features—clinical or investigational—that may in aggregate be both highly predictive and robust to the distributional heterogeneities commonly observed in real-world healthcare data. Mortality risk distributed across multiple investigational modalities, driven by non-linear interactions between remote variables, remains unquantified.

Recent advances in complex modelling now permit a different approach. We can move beyond simple, unimodal, low-dimensional models to complex, multimodal, high-dimensional models that integrate rich information acquired during routine care [12]. This allows us to quantify the benefit—evaluated on out-of-sample data—of flexibly integrating information from multiple sources compared with simpler, unimodal models. If this approach is shown to achieve higher predictive fidelity—if we discover a multimodally distributed predictive signal—an efficient strategy for improving survival prediction would be to deploy flexible multimodal models on existing, routinely collected clinical data. Crucially, introducing this strategy requires only algorithmic and digital innovation—no change to existing care pathways—whereas the discovery of biologically new predictive markers is constrained by the long timelines needed to validate them, the cost of new instruments and assays, and disruption to established clinical management.

Here, we study a large, consecutive, unselected, fully inclusive cohort of older patients acutely admitted to a single acute general hospital, with the following complementary aims: (a) to quantify the predictability from routinely acquired multimodal clinical data of death within 2 years, (b) to compare the performance of predictive models varying in input modality and dimensionality, (c) identify candidate mechanisms of increased mortality, and (d) establish the foundations of a readily deployable clinical tool for predicting all-cause mortality in unselected older patients admitted to acute hospitals, to be fully developed in future large-scale, multi-centre studies. We focus on demographics, primary diagnosis, and cognitive status as simple, readily available clinical descriptors, and routine blood tests and cranial imaging as rich yet objectively quantifiable and reproducible descriptors of physiology and anatomy commonly available in our target population.

## Methods

### Study design and participants

**Fig. 1:**
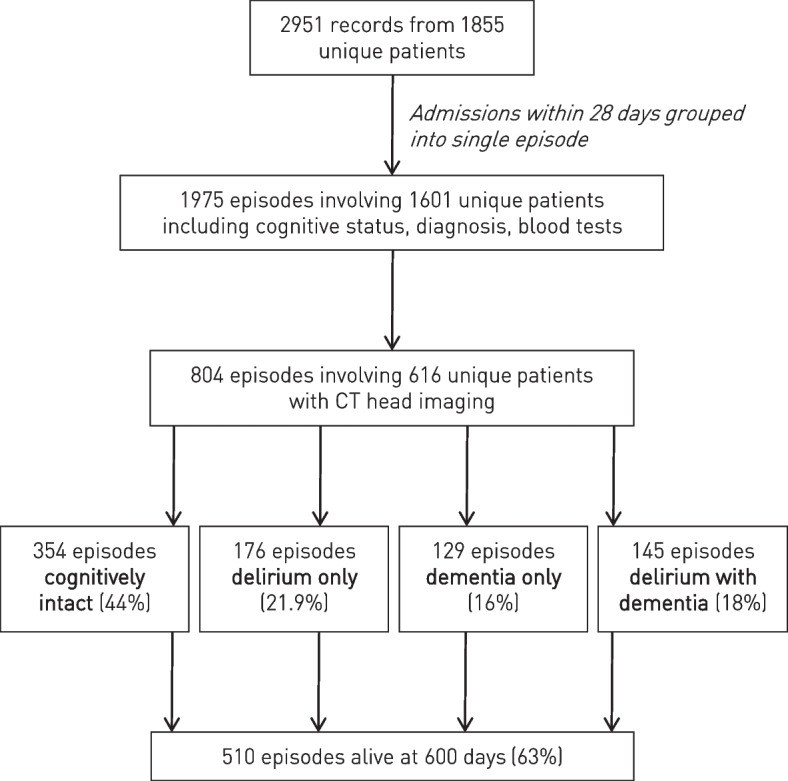
Flowchart of the patient cohort. Routine clinical data from consecutive, unselected patients acutely admitted under the UCLH geriatrics service and evaluated for their cognitive status was linked to admission episodes, associated investigations, and mortality within 600 days

The full study cohort consisted of 2951 consecutive admissions to the acute geriatrics service at University College London Hospital (UCLH) between March 2015 and March 2017 evaluated in the course of an unselected audit of the service. The cohort was drawn from all patients over the age of 70 admitted with any acute general medical problem: the indication for entry into the acute geriatrics service at UCLH. The cohort excluded patients whose admission diagnosis was surgical, or those directly admitted to the intensive care unit. Each patient was reviewed by a consultant geriatrician within 24 h of hospital admission and clinically classified as having (i) delirium only; (ii) dementia only; (iii) delirium superimposed on dementia; or (iv) no or minimal cognitive impairment, from the medical notes and bedside clinical assessment. Admissions were considered as a single episode if the patient was readmitted within 28 days of the prior discharge date. We linked contemporaneous admission information to this clinical dataset, laboratory and imaging investigations, corresponding as closely as possible to the index admission (laboratory results within 48 h of admission; non-contrast CT head imaging performed within four weeks of admission date). The full complement of variables was available for 804 admission episodes involving 616 unique patients (see Fig. 1). The primary diagnosis of each patient was coded as a chapter header of International Classification of Diseases ICD-10. Each patient’s mortality status and date of death were recorded on 24 December 2018 through the hospital vital statistics database (Carecast, GE Healthcare). The study has ethical permission for the analysis of irrevocably anonymized data gathered in the course of routine clinical care. Our reporting adopts the TRIPOD reporting framework.

### Haematological and biochemical investigations

Routinely performed blood tests with coverage of at least 75% of the population—full blood count differentials, red cell distribution width, urea, creatinine, glomerular filtration rate, alanine transaminase, alkaline phosphatase, bilirubin, albumin, potassium, C-reactive protein—were linked for each admission. Where there were multiple values, we used both the chronologically indexed first value and the mean and standard deviation for the rest of the admission. Where only one test was performed, first and mean were identical and standard deviation was zero. This procedure yielded a set of 78 variables capturing both static and dynamic changes in each test. Distributions were visually examined, transformed where appropriate, and clipped to enclose values within 99% of the density of the underlying distribution. Missingness is reported in Additional file 1: Table S1.

Clinical investigations are generally guided by prior, clinically informed belief. To capture the effect of such ‘intention to investigate’, five levels of investigative intention combined with obtained values were defined for each test: (1) investigation performed or not performed (one binary variable); (2) counts of investigation performed over the first 48 ours (one real-numbered variable); (3) investigative intention level 1 and the first test value (two variables); (4) investigative intention level 2 and the mean test value (two variables); and (5) the first test value, mean, and standard deviation (three variables).

Data were modelled at different investigative intention levels to quantify the relative predictive content of the intention to investigate vs the actual test values thereby obtained.

### Cranial imaging

Non-contrast CT imaging of the head performed within 4 weeks of admission for any indication was linked to each patient episode. Each image was processed within an SPM-based (https://www.fil.ion.ucl.ac.uk/spm/) pipeline that included, in order, rigid-body realignment to Montreal Neurological Institute (MNI) space, resampling to 1 mm^3^ isotropic resolution, and non-linear unified spatial segmentation and normalisation to MNI space based on a CT-optimised extension of SPM’s unified segmentation and normalisation routine [13], employing a custom, CT-specific atlas of both intensity and spatial distributions [14] (https://github.com/WCHN/CTseg). The unified segmentation and normalisation approach enables robust segmentation of tissues even in the presence of focal pathological changes, which are implicitly modelled as outliers. The presence and nature of any pathology was not explicitly modelled for the following reasons. First, the diversity of pathological appearances in this population—spanning chronic vascular, degenerative, benign neoplastic, metabolic, and traumatic changes commonly comorbid with acute medical admission—is too wide to be successfully captured at moderate data-scales. Second, leaving diverse variation unmodelled can only reduce predictive performance—our primary task—not spuriously enhance it. Third, our objective is not to create an optimal predictive model but enable a meaningful comparison of model flexibility and input dimensionality. Fourth, deploying an array of disease-specific models would greatly complicate the image analysis, introducing potential dependence on methodological specificities that would limit generalizability.

The output of the pipeline for each patient was two sets of probabilistic tissue segmentation maps of grey matter, white matter, cerebrospinal fluid, skull, and meninges/soft tissue: one native and one non-linearly registered to MNI.

Summary statistics of the volumes of each tissue compartment were derived by thresholding each native-space compartment at > 0.5 and summing the result. Total intracranial volume was quantified as the sum of white matter, grey matter, and cerebrospinal fluid volumes; degree of atrophy, as the sum of grey and white matter divided by total intracranial volume.

Sets of downsampled, signal-optimised, high-dimensional representations of each non-linearly registered compartment were created by cubic resampling of each compartmental image at 5 mm isotropic resolution and extracting all voxels meeting the following criteria: tissue probability > 0.5 and voxel-wise probability variance across the cohort > 0.01. These representations were used as input to the predictive models.

### Predictive modelling

Predictive models for 600 days post-admission mortality were constructed with the gradient boosting machines-based algorithm XGBoost [15]. The choice of algorithm was motivated by the combination of robustness, flexibility, data efficiency, and optimisability given the scale of available data. To quantify the value of increased dimensionality, we estimated an array of models incrementally increasing in number and range of input variables: (1) age and sex (two variables); (2) primary diagnosis, age and sex (17 variables); (3) cognitive status, age and sex (four variables); (4) primary diagnosis, cognitive status, age, and sex (19 variables); (5) bloods, primary diagnosis, cognitive status, age, and sex (91 variables); (6) CT intracranial, primary diagnosis cognitive status, age, and sex (5367 variables); (7) CT extracranial, primary diagnosis, cognitive status, age, and sex (12989 variables); (8) CT whole brain, age, and sex (18399 variables); (9) CT whole brain, bloods, primary diagnosis, cognitive status, age, and sex (18494 variables); (10) CT whole brain, primary diagnosis, cognitive status, age, and sex (18422 variables). The target outcome for all models was survival at 600 days from admission.

The data were randomly split into training (70%) and testing (30%) partitions, stratified by 600-day mortality outcome. Where multiple CT images were obtained in the same admission episode, the first image was always used. The test partition contained unique patients only. XGB models were trained and optimised using tenfold cross-validation from the training partition only with 600-day mortality and the area under the receiver operating characteristic curve (AUROC) as the evaluation metric. A manually targeted grid search followed a random initial parameter grid search to optimise model hyperparameters (number of estimators, maximum depth, minimum child weight, learning rate, gamma, subsample, column sample by tree (Additional file 1: Table S2). The best performing fold hyperparameters as defined by maximal AUROC were used to quantify performance on held-out test data, evaluated through ten-fold cross-validation of the test set only. For completeness, area under the precision-recall curves (AUPRC) are also provided, without model retuning to that objective. SHapley Additive exPlanations (SHAP) values for the top twenty most contributory non-imaging features were derived from the best performing model to illustrate comparative non-anatomical feature weighting in the fitted model. Model calibration, decision threshold curves, and performance variation with age and sex, are evaluated for the best model. No imputation of any missing data was necessary: XGBoost allows missing values to be modelled explicitly.

### Anatomical inference

To understand the anatomical patterns driving the imaging contribution to model fidelity, we sought to identify linear and non-linear voxel-wise associations with the target outcome. To identify linear relations, we performed standard voxel-based brain morphometry of grey matter, white matter, soft tissue, and bone compartments across separate models, all implemented in SPM. At each voxel, the corresponding tissue concentration—the dependent variable—was entered into a multiple regression with survival, age, sex, delirium status, dementia status, degree of global atrophy, and total intracranial volume as independent variables. After model estimation, one-tailed *t*-tests were performed on the regression coefficients with the resultant SPMs interpreted at a *p* < 0.05 family-wise error corrected threshold and displayed at *p* < 0.001 uncorrected to convey the full spatial extent of anatomical association. To identify potentially non-linear associations captured by the XGBoost model, its feature importances, indexed by ranked Gini impurity, were projected back into MNI space for anatomical visualisation.

### Code and data availability

The code employed in this study and derived imaging maps are available from the corresponding authors on request by email. The source data is not available for dissemination under the terms of ethically approved access owing to concerns about potential reidentification in the context of high-dimensional data.

## Results

Over the study period, 2951 admission episodes with established cognitive status and primary diagnoses were recorded from 1855 unique patients. Following grouping of multiple admissions within a 28 day interval as a single episode, 1975 admission episodes, from 1601 unique patients, were defined with linkage to at least one complete set of blood tests within the first 48 h of admission. Across the entire cohort, 804 admission episodes involving 616 unique patients could be linked to a CT head 28 days before or after the day of admission. A flowchart of the cohort definition is provided in Fig. 1.

The mean age was 84.5 years. Cognitive status determined during the admission episodes was distributed as follows: 44% were cognitively intact, 16% had a diagnosis of dementia alone, 21.9% delirium alone, and 18% delirium superimposed on dementia. A total of 36.6% of admission episodes resulted in death within 600 days (Table 1).

**Table 1:** Summary of complete admissions cohort

	Alive at 600 days (***n*** = 510)	Deceased at 600 days (***n*** = 294)	***p*** value
**Age (mean, 95% CI)**	83.7 (69.98–97.42)	85.9 (72.38–99.42)	< 0·01
**Women (%, 95% CI)**	27.6 (23.72–31.48)	16.8 (12.53–20.68)	< 0·01
**Cognitive status (%, 95% CI)**			0·02
**Cognitively intact**	46.70 (42.37–51.03)	39.77 (34.18–44.10)	
**Delirium only**	20.35 (16.86–23.84)	24.86 (19.92–28.35)	
**Dementia only**	17.03 (13.77–20.29)	14.36 (10.35–17.62)	
**Delirium + dementia**	15.93 (12.75–19.11)	22.10 (17.36–25.28)	
**Brain volume (mls, 95% CI)**	917 (742–1091)	908 (716–1100)	0·20
**Atrophy (%, 95% CI)**	81.4 (77.28–85.52)	81.1 (77.18–85.02)	0·35

Mean values are presented for continuous variables, with 95% confidence intervals in brackets. The *p* values reported in the last column are derived from chi squared tests for categorical variables and two sample *t*-test for continuous variables. The value for cognitive status assumes ordinal progression in cognitive decline across the categories itemised beneath

### Mortality prediction from multimodal data

**Fig. 2:**
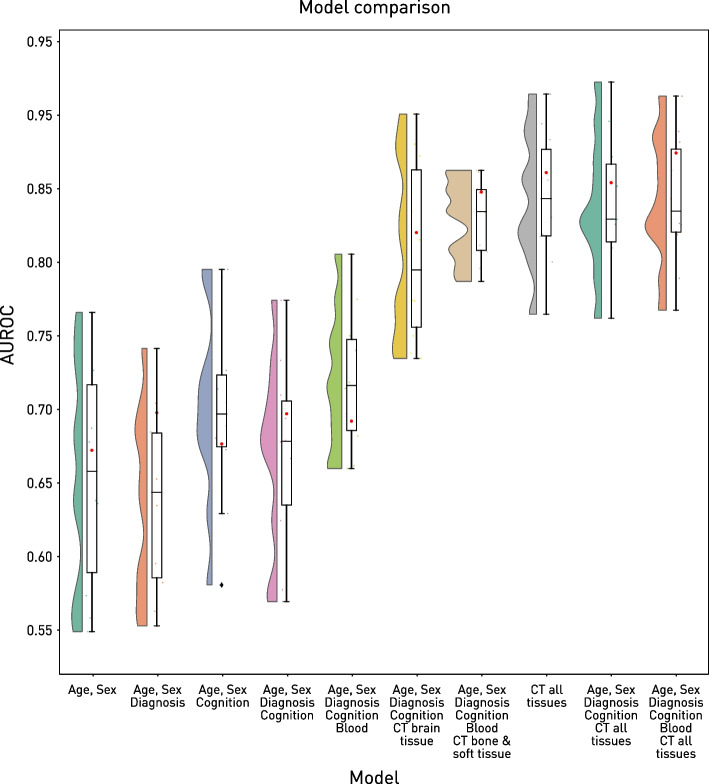
The effect of feature number and modality on predictive performance. For each of a set of incrementally complicated models, the distribution of the area under the receiver operating curve (AUROC) values obtained from cross-validation is presented as a violin and box plot, and the single AUROC on the held-out test set is plotted as a red circle. Note the marked increase in performance associated with the addition of information, especially from cranial imaging

Baseline XGB predictive models of age and sex achieved only a modest AUROC of 0.672 on out-of-sample test data (Fig. 2). There was no marked benefit from the addition of clinical features: cognitive status (AUROC = 0.677) or primary diagnosis (AUROC = 0.698), either alone or together (AUROC = 0.697). The addition of blood tests to the full clinical model yielded no improvement over the baseline (AUROC = 0.692) that was not explicable by the intention to investigate alone (Additional file 1: Figure S1).

A substantial jump in predictive performance was observed with the addition of imaging data to the full clinical model. The combination of intracranial imaging with demographic and clinical features yielded an AUROC of 0.82, substantially higher than both baseline and demographic and clinical data alone. The use of extracranial features produced comparable performance (AUROC = 0.848) in otherwise identically specified models. Intracranial and extracranial compartments alone (AUROC = 0.861) or in combination with demographics and clinical features (AUROC = 0.854) yielded similar performance. The highest performing model included the widest selection of inputs—demographics, clinical features, blood tests, intracranial and extracranial CT*—* exhibiting an AUROC of 0.874. The variability in performance is captured by the cross-validation distributions on the training set (Fig. 2). Analysis of the area under the precision-response curve yielded an essentially identical pattern (Additional file 1: Figure S2).

**Fig. 3:**
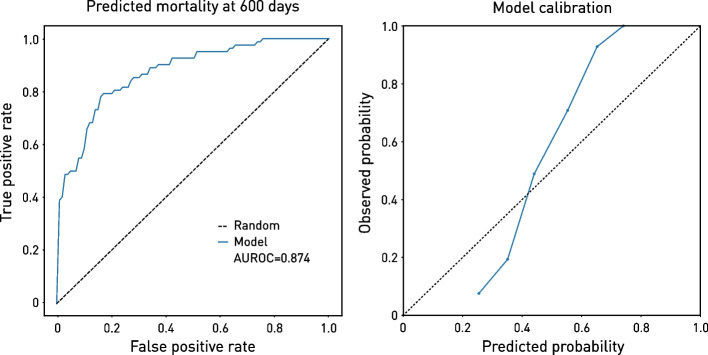
Best model performance characteristics. Receiver operating characteristic curve on the held-out test data of the highest performing model (left), which included the widest selection of inputs—demographics, clinical features, blood tests, intracranial and extracranial CT. Model calibration is shown on the right

The best model exhibited good calibration (Fig. 3) and threshold diagnostics (Additional file 1: Figure S3), as well as reasonably equitable performance across age and sex (Additional file 1: Figure S4). Note, however, that this does not define the limit of achievable performance: our objective here is to demonstrate the predictive potential of the approach rather than to maximise it, for which a larger study is appropriate.

### Haematological and biochemical correlates of mortality

**Fig. 4:**
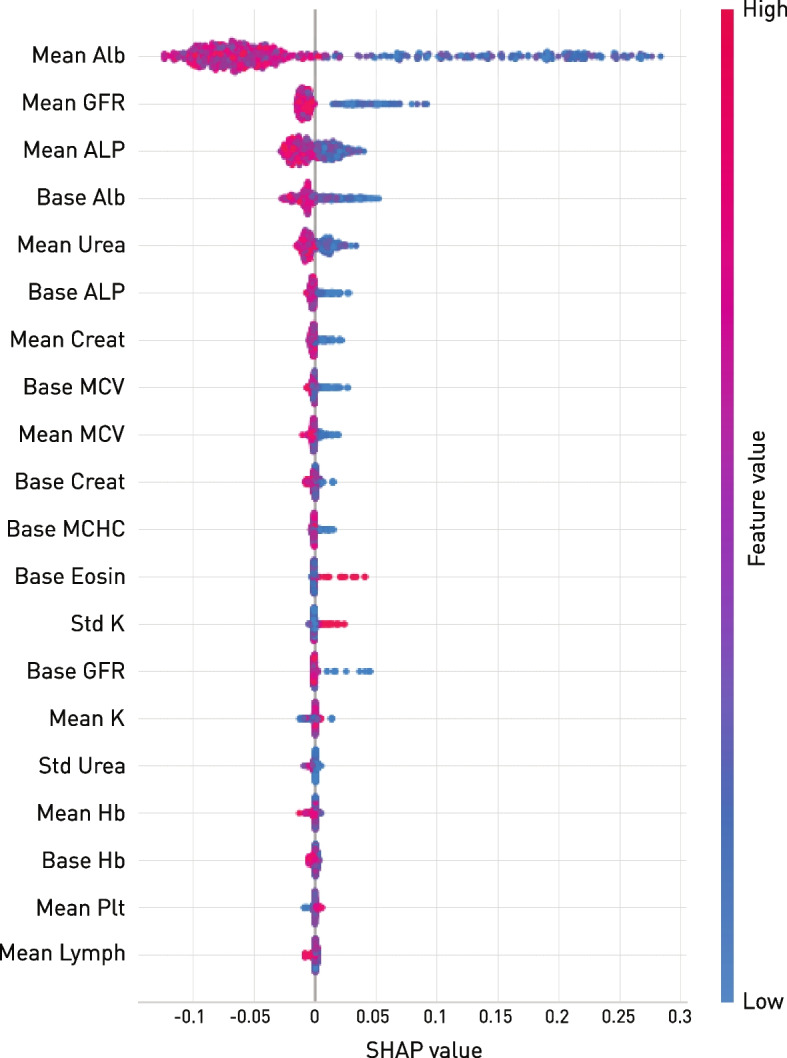
SHAP values of the top twenty haematological and biochemical contributions to the best predictive model. A higher SHAP value indicates a higher risk of death for the feature value indicated in the colour map. Note that lower albumin, glomerular filtration rate, alkaline phosphatase, urea, and creatinine, and microcytic haematological features offer the greatest support to the model, in line with sensitivity to the somatotrophic state

The SHAP values of the top twenty non-imaging features making the greatest contribution to the best predictive model highlighted renal function and plausible markers of the somatotrophic state: glomerular filtration rate, albumin, alkaline phosphatase, urea, creatinine, and red cell indices (Fig. 4). Note that these features are drawn from the multimodal model, where collinearities—e.g. between brain morphology and age—will inevitably influence the ranking: this is not a test of the independent association of each feature, only an index of its contribution to the model’s performance. Owing to their number and density, the imaging features are presented anatomically in the next section.

### Anatomical correlates of mortality

**Fig. 5:**
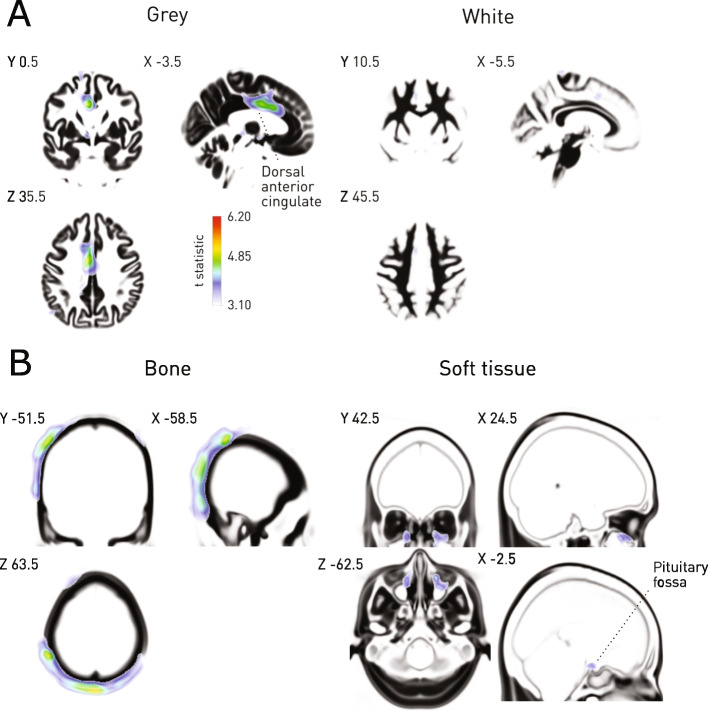
Cranial and extracranial correlates of mortality. **A** Voxel-based morphometry of grey and white matter concentrations shows an association with dorsal anterior cingulate grey matter concentrations. The *t* statistic map for the voxelwise contrast ‘survived’ > ‘deceased’ is shown overlaid on the mean of the corresponding tissue compartment, thresholded at *p* < 0.001 uncorrected, with the *p* < 0.05 FWE threshold shown on the colourbar. **B** Voxel-based morphometry of bone and soft tissue compartmental concentrations shows an association with cranial bone density. The *t* statistic map for the voxelwise contrast ‘survived’ > ‘deceased’ is shown overlaid on the mean of the corresponding tissue compartment, thresholded at *p* < 0.001 uncorrected, with the *p* < 0.05 FWE threshold shown on the colourbar

Voxel-wise mass univariate models of mortality revealed multiple loci of linear association distributed across the intracranial and extracranial compartments (Fig. 5). Intracranially, the most prominent associations were seen in the dorsal anterior cingulate grey matter. Extracranially, widespread modulation was seen in the vicinity of the parietal and occipital bones, with further loci not surviving conservative multiple comparisons correction observed within the sinuses, and the region of the pituitary fossa.

**Fig. 6:**
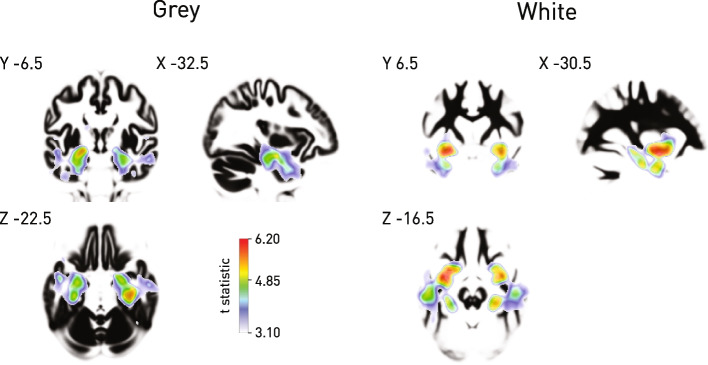
Grey and white matter correlates of dementia. Voxel-based morphometry of grey and white matter concentrations derived from cranial CT shows strong modulation of medial temporal grey and white matter typical of the most common clinical subtype. The *t* statistic map for significantly reduced concentrations is shown overlaid on the mean of the corresponding tissue compartment, thresholded at *p* < 0.001 uncorrected for multiple comparisons to enable visualisation of the full extent of modulation, with the *p* < 0.05 FWE threshold shown on the colourbar

A diagnosis of dementia was strongly associated with loss of medial temporal grey and white matter typical of the most common clinical subtype, confirming the sensitivity to pathological changes of our analytic approach, despite the biological and instrumental heterogeneity inevitable in an unselected, clinical dataset, and the use of CT rather than MR imaging (Fig. 6).

### Anatomical features of predictive importance

**Fig. 7:**
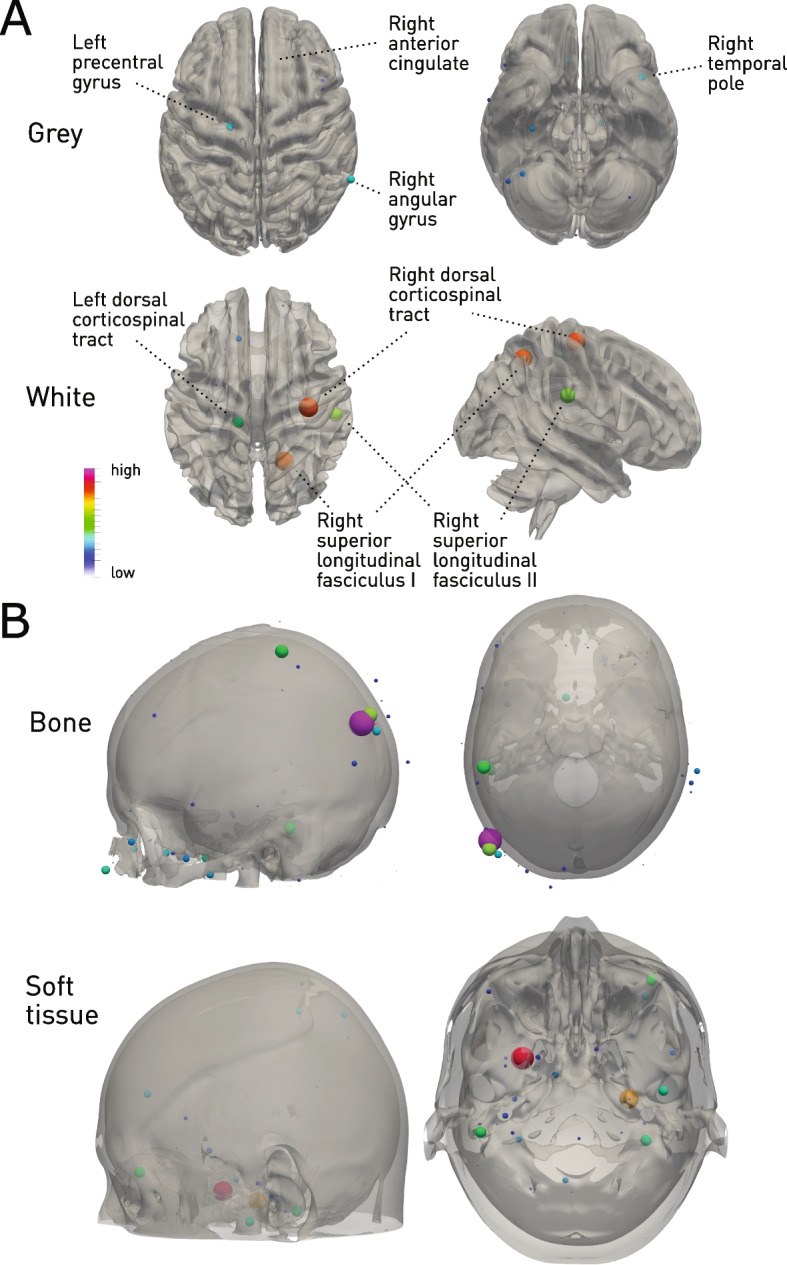
Cranial and extracranial predictors of mortality. Glyph-based representation of voxels with high feature importance in the best-performing XGBoost model, overlaid on surfaces extracted from the corresponding tissue compartment. **A** Left precentral gyrus, right anterior cingulate, right angular gyrus and right temporal pole, the region of the dorsal corticospinal tract, and the right superior longitudinal fasciculus are highlighted. **B** Extracranially, parietal bone was highlighted, and diffusely distributed soft tissue. Note that laterality differences are difficult to interpret here as any collinearity of dependence arising from hemispheric symmetry would result in down-weighting of redundant features: this is an inevitable feature of flexible, high-dimensional discriminative models

Projection of the anatomical feature importances derived from the best performing XGB model showed a widely distributed pattern of dependence (Fig. 7). Intracranially, left precentral gyrus, right anterior cingulate, right angular gyrus and right temporal pole, the region of the dorsal corticospinal tract, and the right superior longitudinal fasciculus were highlighted. Extracranially, parietal bone was highlighted as in the linear models and diffusely distributed soft tissue. Note that a collinearity of dependence as might arise from hemispheric symmetry would be handled by the model by down weighting redundant features: this precludes strong interpretations of laterality here and is an inevitable feature of flexible, high-dimensional discriminative models.

## Discussion

Examining a large, unselected cohort of acutely hospitalised elderly patients evaluated for the presence of acute or chronic cognitive impairment and imaged with cranial CT, we have quantified the predictability from routinely acquired multimodal clinical data of death within 2 years, compared the performance of predictive models varying in input modality and dimensionality, and characterised the distribution of multimodal biological factors predictive of mortality identify. Our findings provide the foundations for developing a readily deployable clinical tool for predicting mortality in older patients within future large-scale, multi-centre studies. Here, we consider the merits and demerits of the proposed approach our analysis suggests.

### The predictability of mortality from routine clinical data

Employing strictly out-of-sample evaluation of performance, we showed that 600-day mortality is predictable with high fidelity—AUROC 0.858—from the combination of basic clinical data with routine investigations. This suggests the presence of stronger predictive signals than are harnessed by current mortality prediction models—ranging from 0.5 1[16] to 0.7 8[11]—especially when applied to patients aged 75 and over [11, 17]. Of note, the signal here is grounded primarily in fundamental, quantitative biological characteristics of the patient rather than the circumstances of their care—such as mode and specialty of admission—minimising the risk of poor generalisability across other healthcare systems observed for higher-performing scores such as HOMR [9, 11, 18]. A critical contribution is made by intra- and extracranial characteristics captured by CT imaging whose quantitative nature renders them more reproducibly identifiable than those based on non-quantitative imaging such as MRI. Robustness to clinical and demographic variation naturally requires evaluation over other, widely dispersed cohorts, but the level of observed fidelity motivates further exploration of such predictive models embeddable within the existing clinical pathway without disruption to established care.

### Predictive model complexity

Though desirable for their intelligibility and generalisability, simple predictive models are constitutionally incapable of integrating information distributed across multiple interacting factors. Where, as here, the causal field is plausibly wide and densely interdependent, models of greater dimensionality and flexibility are required. Exploiting the flexibility and robustness of gradient boosting machines, we have shown that escalating model dimensionality is rewarded by more accurate predictive performance—quantified out-of-sample. This indicates the presence of distributed, possibly interacting, factors that collectively strongly predict mortality even if they may be only weakly predictive in isolation. The scale of the informative dimensionality—thousands of variables—suggests room for improvement with more data and finer model architectural tuning. Note that any substantive increment in predictive fidelity is valuable for a model applied at the individual patient level—an imperfect model here can never be too accurate. The greater sensitivity of complex models to distributional shift remains a challenge but is no longer an insuperable obstacle: it is addressable algorithmically and through expanding the scale and diversity of modelled data (e.g. [19–21]).

Though imaging provides the greatest incremental boost in fidelity, integration of multimodal data within a unified predictive model yields the best performance. Given the comparative ease of modelling routine clinical and non-imaging investigations, their incorporation would be justified even by small improvements in model prediction. Whether or not the addition of any modality increases model performance nonetheless remains an empirical question that depends both on the informativity of a given modality and the cross-modal redundancy of the predictive signal. The use of modalities, such as brain imaging, that provide a rich, distinctive, detailed perspective on biology reduces the likelihood of redundancy even if it always remains a possibility. A degree of redundancy is in any event desirable in providing some insurance against the missingness—both during model training and at test time—common in real-world clinical environments.

### Possible mechanisms of increased mortality

Our mass-univariate analyses of haematological, biochemical, and especially imaging features point to a unifying association of mortality with sarco- and osteopenia, with especially striking modulation of parietal and occipital bone and cranial soft tissue. Sarcopenia, defined as the progressive generalised loss of skeletal muscle, is associated with increased mortality risk [22], potentially mediated via impaired mobility, falls, and respiratory complications [23, 24]. Similarly, osteoporosis and the increased fractures are well recognised to be associated with increased mortality risk: neck of femur fractures are associated with one-year mortality of around 30 %[25]. Albumin is commonly acknowledged to be a poor marker of nutritional status, particularly in the acute setting: its predictive contribution plausibly represents altered hepatic synthesis in favour of acute phase proteins, a potential marker of proinflammatory acute illness. The contributions of urea and alkaline phosphatase are consistent with modulation by sarcopenia and bone density respectively.

Within the brain, striking modulation of medial frontal cortical areas implicated in voluntary motor behaviour and autonomic function—principally the anterior cingulate—may be explained by the potential impact of dysfunction in either domain. The potential importance of motor function is reinforced by the enhanced multivariate feature importance of voxels falling within the primary motor cortex and the corticospinal tract. Note that these changes may be explained not only by brain pathology—such as small vessel disease—that either directly or indirectly reduces grey matter concentrations locally but potentially by neural adaptation to long term reduced mobility of non-neural origin. The involvement of areas with dense (angular gyrus) or remote (temporal pole) connectivity may also reflect differential rates of length-dependent degeneration in white matter and the grey matter it connects. Further exploration of these biologically intelligible patterns—yet to be interrogated by others in this population—is merited.

### A multimodal index of frailty

A predictive model with high fidelity at the individual level has the potential to support clinical decision-making. Here quantifying the risk of death early on in the course of a hospital admission enables proportionate pre-emptive action—by both patients and clinicians—to minimise it. Grounding an index of frailty in multimodal signals in principle renders it more robust to incidental variations by broadening its evidential support, and potentially widens the field of manipulable factors critical in any one patient, permitting more closely individuated interventions. Adapting the predictive model to absorb greater population heterogeneity, and incorporating machinery for causal inference, require further algorithmic development with larger-scale data that this proof-of-concept now justifies. Crucially, since routine clinical and investigational data models appear to be sufficiently powerful here, real-world implementation of a decision-support tool does not require any changes to clinical pathways, substantially lowering barriers to adoption. Indeed, it may be argued that clinicians have a moral duty to maximise the guiding intelligence extracted from the data they obtain from patients, often at individual, and always at institutional, cost.

Some may be tempted to use models of this kind to guide withdrawal of treatment. Where a plausible causal chain—and its clinical modifiability—are not established, such use cannot be justified and should be proscribed, not just in the case of complex, high-dimensional models but any predictive model. Indeed, demonstrating the predictability of individual variations in risk provides a strong argument against any generic approach to treatment withdrawal.

A high-fidelity individual index of frailty also has applications in stratifying patients in observational and interventional research studies, where unmodelled structured variability could otherwise conceal or distort inferred effects. Furthermore, multimodal models may reveal heterogeneities between subpopulations exhibiting the same risk, suggesting potential differences in causation that would confound inferences unless explicitly modelled. Attention to heterogeneity is paramount in the older patient, where the multiplicity and diversity of observed pathologies is high. Identifying subpopulations of especially elevated risk facilitates the identification of mechanistic factors of potential interventional value concealed by noise or non-linearities. Although highlighting sarcopenia and the integrity of motor brain areas provides actionable targets, downstream studies are needed to quantify the clinical utility of any intervention.

We do not advocate scanning patients purely for prognostic purposes, only using CT data acquired commonly enough for a model based on it to be widely applicable. If the fidelity of the approach is established over larger scale, multi-site studies, and clinical utility is demonstrated—in guiding preventative interventions, for example—a case could be made on the specific balance of risk and benefit. But since the model’s support is drawn from those in whom CT is clinically indicated, transfer to the wider population would need careful evaluation. The increasing digital maturity of radiological systems and their widespread integration with electronic healthcare records make this potentially feasible at substantial scale and with broad inclusivity.

### Strengths and limitations

The synthesis of multimodal signals spanning demographics, clinical features, blood tests, and CT imaging data is unique amongst prognostic models in this population. The use of objectively quantifiable features derived only from routinely collected data without the need for pre-admission information or potentially subjective clinical assessment is a central strength, promoting generalisation across healthcare systems and enabling implementation without disrupting established pathways. Model development and out-of-sample validation on one of the largest unselected cohorts of older patients evaluated for acute illness in frail patients is grounds for confidence in the robustness of the findings.

An array of limitations should be noted. Though the largest of its kind, this is a retrospective study validated on held-out but retrospective data from the same institution: generalisability over time and location must be established in subsequent studies. In keeping with all observational analyses of routine clinical data, a degree of corruption by (potentially structured) missingness, acquisition, and documentation errors, and clinical uncertainty is inevitable. Specifically, the diagnoses of delirium and dementia, though made by a senior clinician, were not validated by dedicated instruments or (in the case of dementia) corroborative imaging. Equally, though our cohort is fully inclusive of the clinical stream, only those with CT imaging of the head, carried out for indications individual to each patient, were retained in the analysis. We pursued this approach to maximise ecological validity, replicating the quality of data a real-world institution would naturally see. The impact of potential biases is minimised by the use of sequential, unselected data, enabling inference across all those in receipt of the criteria investigations. We explicitly quantify the effects of intention to investigate in relation to individual blood tests, finding it to contain negligible predictive signal. Institutional-level variability in clinical practices could, of course, impact generalisation and need exploration in future multi-centre studies. Because it was not available, we do not model the cause of death, only the primary diagnosis on the admission that triggers entry into the cohort. Co-morbidities were omitted for the same reason. There is no reason to expect the distribution of causes of death to differ substantially from that observed for the underlying population, and our focus here is the fact of it rather than its cause. But incorporating cause of death and co-morbidities, in further escalation of model complexity, may well increase performance and ought to be evaluated where possible. Finally, the use models that are high-dimensional in proportion to the number of training instances always presents a risk of overfitting, here addressed by strong regularisation, a modelling architecture relatively resistant to overfitting, and careful evaluation of the model with both cross-validation and a fully held-out definitive test set. It is for this reason that further evaluation, within larger, multi-site studies is recommended here.

## Conclusions

Examining the distribution of a wide array of multimodal predictive factors in older patients acutely admitted to hospital and imaged with cranial CT, we have established the foundations of a multimodal approach to predicting medium-term mortality, operational at the point of admission, and validated for unselected oldest-old patients. Our analysis demonstrates the benefit of using machine learning to enable models incorporating multiple modalities, highlighting the predictive potential of defining ‘multimodal frailty’. Inferences drawn from the prediction model suggest that unimpaired higher motor and autonomic function is associated with medium-long term survival. These results justify further investigation of the application of high-dimensional modelling to predicting mortality in the older patient.

## Supplementary Information

**Additional file 1: Fig. S1.** Intention to investigate effects in blood tests. **Fig. S2.** The effect of feature number and modality on predictive performance. **Fig. S3.** Decision threshold diagnostics for the best multimodal model. **Fig. S4**. Subpopulation performance. **Table S1.** Blood test missingness, ordered by magnitude. **Table S2.** Hyperparameter cross-validation grid search range and XGboost final hyperparameters.

## Data Availability

Group statistical maps are available from the corresponding author on request, but the raw data is not available under the provisions of ethical approval.

## Abbreviations

CT: Computed tomography
MR: Magnetic resonance
SPM: Statistical parametric mapping
AUROC: Area under the receiver operating characteristic curve
AUPRC: Area under the precision-recall curve
FWE: Family wise error
