# Supplementary material for: Predicting mortality in acutely hospitalised older patients: the impact of model dimensionality
Source: BMC Med. 2023 Jan 8;21:10. doi: 10.1186/s12916-022-02698-2 (PMC9827638; doi:10.1186/s12916-022-02698-2)
Supplement: Supplementary file 1 — Additional file 1: Fig. S1. Intention to investigate effects in blood tests. Fig. S2. The effect of feature number and modality on predictive performance. Fig. S3. Decision threshold diagnostics for the best multimodal model. Fig. S4. Subpopulation performance. Table S1. Blood test missingness, ordered by magnitude. Table S2. Hyperparameter cross-validation grid search range and XGboost final hyperparameters. [file 12916_2022_2698_MOESM1_ESM.pdf]

Predicting mortality in acutely hospitalised older patients: the impact of model dimensionality

Additional file

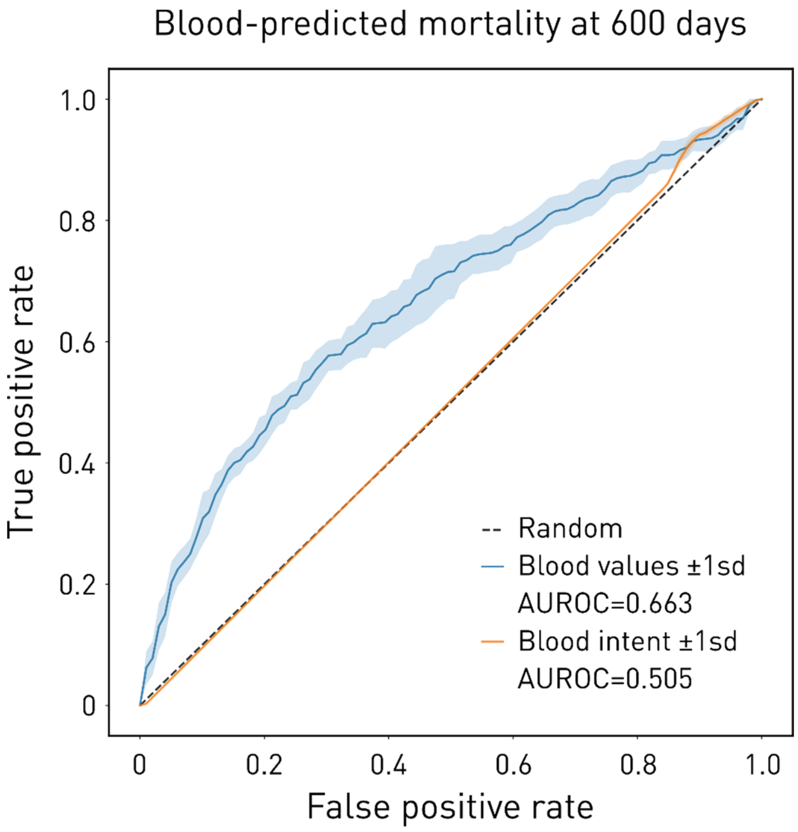

Figure S1. Intention to investigate effects in blood tests. Comparison of the predictive performance of models of the values of blood tests (blue) vs their indication (orange), as revealed by the receiver operating characteristic on the test data (dark colours) with standard deviations from cross-validation (light colours). Note the absence of any evidence that blood predictive signals are carried by the indication rather than by the values themselves.

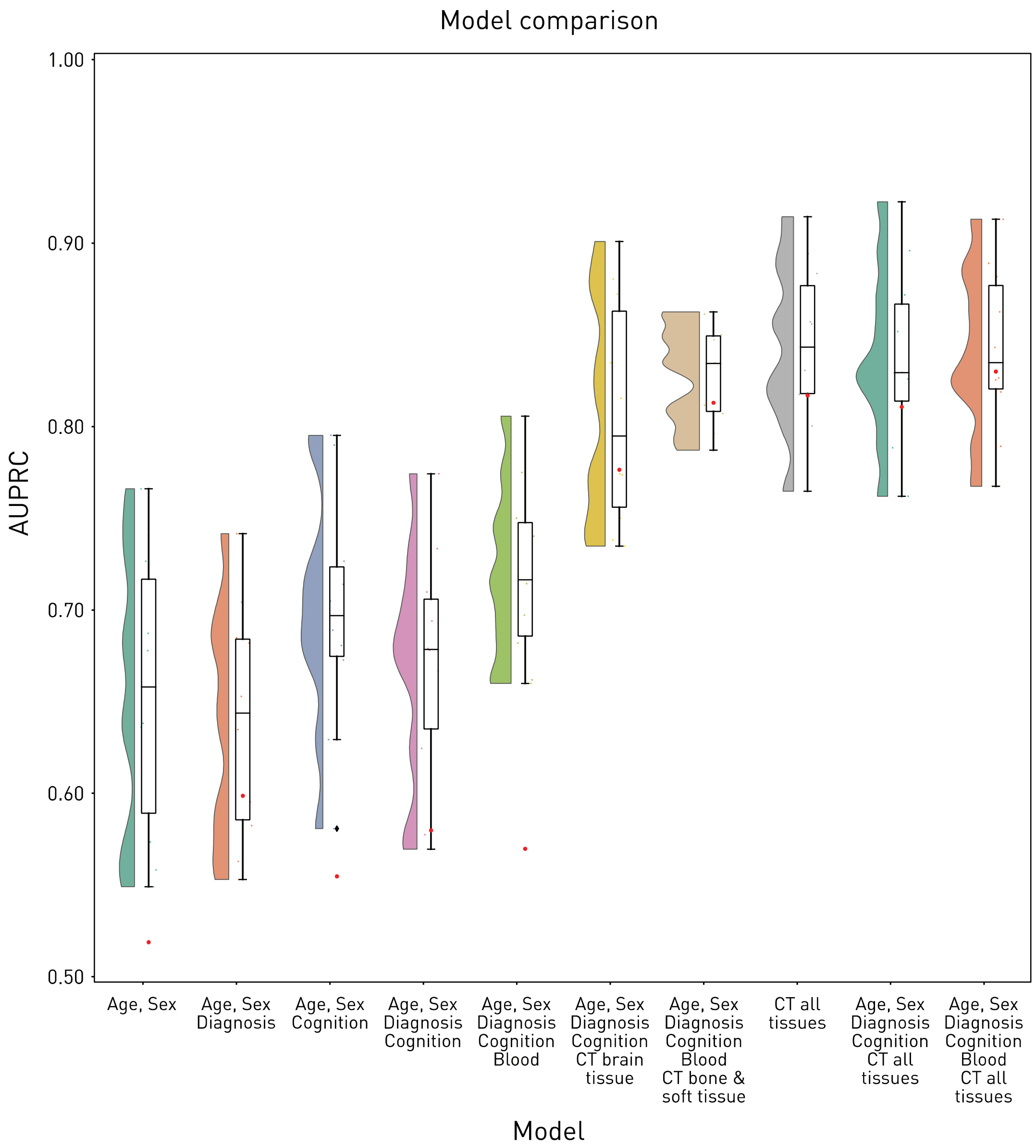

Figure S2. The effect of feature number and modality on predictive performance. For each of a set of incrementally complicated models, the distribution of the area under the precision-recall curve (AUPRC) values obtained from cross-validation is presented as a violin and box plot, and the single AUPRC on the held-out test set is plotted as a red circle. Note the marked increase in performance associated with the addition of information, especially from cranial imaging. Note absolute AUPRC performance is poorer than AUROC in reflection of the models not being tuned on it.

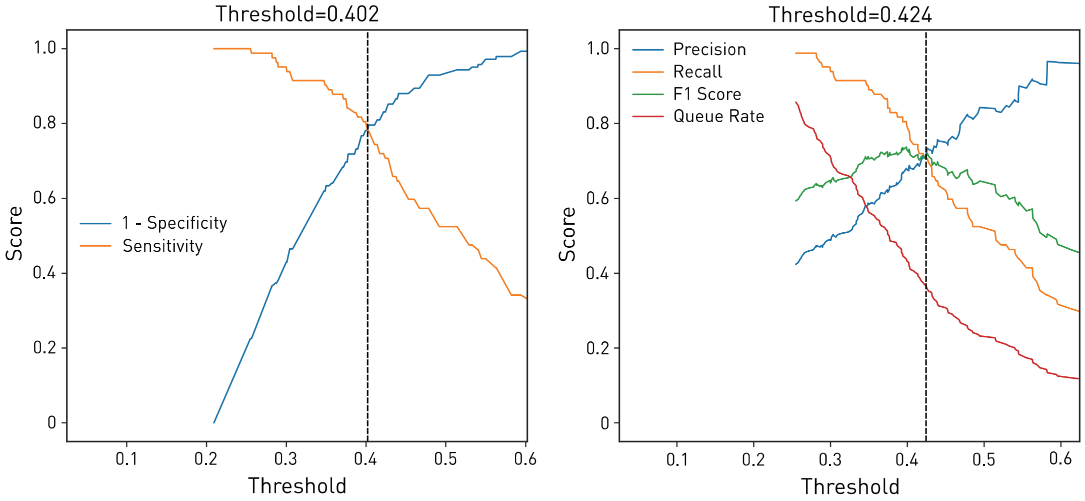

Figure S3. Decision threshold diagnostics for the best multimodal model. Sensitivity, specificity, precision, recall, F1, and queue rate on the test data are shown as a function of the model threshold.

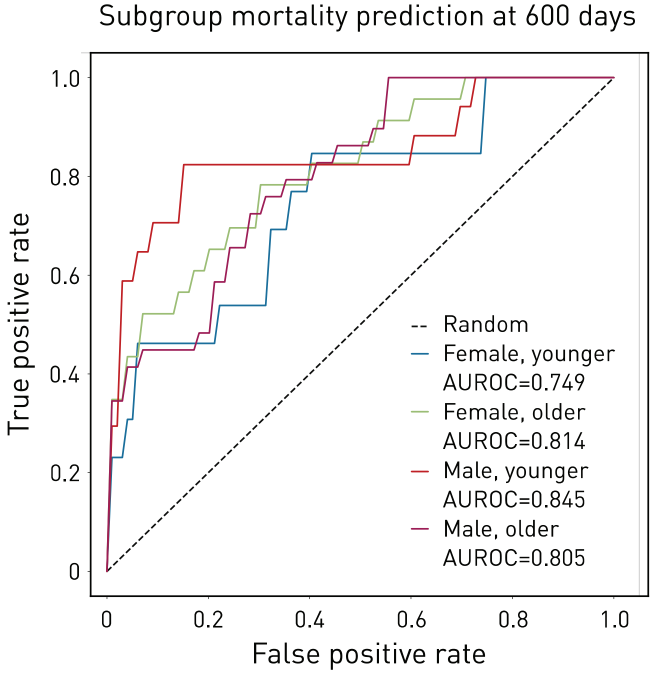

Figure S4. Subpopulation performance. Receiver operating characteristic curves for subpopulations stratified by age and sex. Note no optimisation for equitability has been performed here: this does not represent the limit of equitable performance.

Table S1. Blood test missingness, ordered by magnitude.

Blood test	Missing
Corrected Calcium	25.22%
Prothrombin Time	14.73%
Alanine Aminotransferase	9.42%
Bilirubin	8.30%
Alkaline Phosphatase	7.95%
Albumin	6.68%
Potassium	4.15%
C-Reactive Protein	1.27%
Red Cell Distribution Width	0.66%
Platelet	0.51%
Creatinine	0.51%
Haemoglobin	0.46%
Monocyte	0.46%
Mean Corpuscular Volume	0.46%
Basophil	0.46%
Eosinophil	0.46%
Red Cell Count	0.46%
Lymphocyte	0.46%
Haematocrit	0.46%
White Cell Count	0.46%
Neutrophil	0.46%
Urea	0.35%

Table S2. Hyperparameter cross-validation grid search range (top) and XGboost final hyperparameters (bottom).

Hyperparameter	Grid search range
Early stopping rounds	200
n_estimators	Free
Maximum depth	[3, 5, 7, 9]
Minimum child weight	[1, 3, 5, 7, 9]
Gamma	0 to 1 (in 0.1 intervals)
Subsample	0.6 to 1 (in 0.1 intervals)
Column sample by tree	0.6 to 1 (in 0.1 intervals)

Hyperparameter	Value
Learning rate	0.1
Minimum child weight	1
N estimators	196
Maximum depth	7
Gamma	0
Subsample	0.9
Colsample by tree	0.6
Scale position weight	1
Random state	42
